# Transplanted allogeneic cardiac progenitor cells secrete GDF-15 and stimulate an active immune remodeling process in the ischemic myocardium

**DOI:** 10.1186/s12967-022-03534-0

**Published:** 2022-07-21

**Authors:** Rachana Mishra, Progyaparamita Saha, Srinivasa Raju Datla, Pranav Mellacheruvu, Muthukumar Gunasekaran, Sameer Ahmad Guru, Xubin Fu, Ling Chen, Roberto Bolli, Sudhish Sharma, Sunjay Kaushal

**Affiliations:** 1 Department of Cardiovascular-Thoracic Surgery, Northwestern University Feinberg School of Medicine, Chicago, IL USA; 2 Department of Pediatrics, Ann & Robert H. Lurie Children’s Hospital, Chicago, IL USA; 3 Department of Surgery, University of Maryland School of Medicine, Baltimore, MD USA; 4 Division of Cardiovascular Medicine and Institute of Molecular Cardiology, University of Louisville, Louisville, USA

**Keywords:** Cardiac progenitor cells, Myocardial ischemia, Growth differentiation factor 15, Immunomodulatory properties, T-regulatory cells, Macrophages

## Abstract

**Background:**

Despite promising results in clinical studies, the mechanism for the beneficial effects of allogenic cell-based therapies remains unclear. Macrophages are not only critical mediators of inflammation but also critical players in cardiac remodeling. We hypothesized that transplanted allogenic rat cardiac progenitor cells (rCPCs) augment T-regulatory cells which ultimately promote proliferation of M2 like macrophages by an as-yet undefined mechanism.

**Methods and results:**

To test this hypothesis, we used crossover rat strains for exploring the mechanism of myocardial repair by allogenic CPCs. Human CPCs (hCPCs) were isolated from adult patients undergoing coronary artery bypass grafting, and rat CPCs (rCPCs) were isolated from male Wistar-Kyoto (WKY) rat hearts. Allogenic rCPCs suppressed the proliferation of T-cells observed in mixed lymphocyte reactions in vitro. Transplanted syngeneic or allogeneic rCPCs significantly increased cardiac function in a rat myocardial infarct (MI) model, whereas xenogeneic CPCs did not. Allogeneic rCPCs stimulated immunomodulatory responses by specifically increasing T-regulatory cells and M2 polarization, while maintaining their cardiac recovery potential and safety profile.

Mechanistically, we confirmed the inactivation of NF-kB in Treg cells and increased M2 macrophages in the myocardium after MI by transplanted CPCs derived GDF15 and it’s uptake by CD48 receptor on immune cells.

**Conclusion:**

Collectively, these findings strongly support the active immunomodulatory properties and robust therapeutic potential of allogenic CPCs in post-MI cardiac dysfunction.

**Supplementary Information:**

The online version contains supplementary material available at 10.1186/s12967-022-03534-0.

## Introduction

Due to their anti-inflammatory and tissue reparative properties, cardiac stem cells are being increasing studied as potential allogeneic cell-based therapies to treat various inflammatory diseases, including ischemic heart disease, heart failure, and congenital heart disease [1]. Although transplanted autologous cells induce minimal to no immune responses and have shown efficacy in clinical trials, treatment with autologous cardiac-derived cells is complicated by an additional delay for the preparation of cells from the patient’s cardiac tissue. Additionally, the clinical efficacy of autologous cells can vary significantly from patient to patient due to age differences, disease severity, co-morbidities, and medications [2]. Allogeneic cell therapy circumvents these limitations of autologous cell preparations. The immunosuppressive properties and low immunogenicity of certain allogenic cells contribute to a reduced or weakened immune response compared with other cell types [3].

Allogeneic cardiosphere-derived cells (CDCs; CAP-1002) and bone marrow derived mesenchymal stem cells (MSCs) have been used in Phase II clinical trials for patients with myocardial infarction (MI)/ischemic left ventricular dysfunction and cardiomyopathy [1, 4]. In addition, therapy with autologous c-kit-positive cardiac cells (CPCs) improves cardiac function and structure in animal models of myocardial infarction (MI) and has recently been shown to benefit patients with ischemic heart failure [5, 6]. An important question with these allogeneic cell therapies is whether host immune responses compromise their safety and efficacy. Equally critical for either autologous or allogeneic cell preparations is that their therapeutic benefits appear to be mediated by paracrine factors secreted by the transplanted cells, rather than by differentiation of the transplanted cells into cardiomyocytes or cells of another cardiac lineage [1]. Furthermore, with any cell therapy, there is very low tissue engraftment of the transplanted cells because they are rapidly cleared from the myocardium within a few weeks after injection [1, 7, 8]. The current consensus is that paracrine or endocrine mechanisms trigger endogenous myocardial reparative/regenerative processes that do not depend on the sustained presence of the transplanted cells [1, 8]. However, the host immunological responses to either the transplanted cells or their secreted paracrine factors remain unclear.

Innate and inflammatory immune responses play a pivotal role in ischemia-induced cardiac damage and repair processes by triggering a cascade of events with the aim of healing the injured tissue [9]. First, neutrophils and monocytes/macrophages infiltrate the tissue to remove necrotic debris. This is followed by resolution of inflammation, fibroblast activation, replacement fibrosis, and scar tissue formation [10]. Modulation of the immune response during MI is thought to offer a therapeutic benefit by expediting myocardial recovery [11]. Mounting evidence suggests that MSCs modulate the immune response by inhibiting cytotoxic T cells and increasing proliferation of regulatory T cells (Tregs) [12]. Additionally, MSCs induce angiogenesis by polarizing macrophages toward the M2 phenotype [13]. Furthermore, CDCs have been shown to promote immune tolerance by activating programmed death ligand 1 (PD-L1), an immune checkpoint modulator that suppresses excessive immune activation [14]. These critical immune pathways triggered by transplanted cells have been recently questioned by one study showing that transplanted CPCs failed to reduce inflammation, and that when inflammation was stimulated by even transplanted dead CPCs a similar functional recovery was observed in a murine MI model [15]. Thus, additional investigation is warranted to further define the response of immune cells to transplanted cells and its mechanism(s).

We have shown previously that CPCs secrete Growth Differentiation Factor 15 (GDF15) [8], a cytokine produced in response to mitochondrial stress thought to contribute to adaptive homeostatic changes in response to tissue damage [16]. GDF15 has also peripheral anti-inflammatory/immunomodulatory and cardioprotective effects [17–21]. We hypothesized that transplanted allogeneic CPCs are immunomodulatory and exert cardioprotective effects similar to those observed with autologous CPCs. We therefore investigated immunologic responses to syngeneic, allogeneic, and xenogeneic CPCs in immunologically divergent species without immunosuppressive therapy. Additionally, we investigated the fundamental mechanism by which CPCs promote recovery of the injured heart. We found that CPCs transplanted in the post-ischemic heart secrete GDF15 into the myocardium, promoting T-reg and macrophage polarization through a previously recognized GDF15 receptor CD48 [21] on T cells. GDF15 secretion inactivates NF-κB in local T-regs present on the myocardium, facilitating polarization toward the cardioprotective anti-inflammatory M2 phenotype.

## Materials and methods

### Rat c-kit-positive cardiac cells

Rat c-kit-positive cardiac cells (rCPCs) were isolated from male Wistar-Kyoto (WKY) rats (6–8 weeks of age) as described previously [16]. Briefly, rat hearts were isolated and perfused via the aortic root with phosphate-buffered saline (PBS), followed by a solution of collagenase (128 units/ml) and hyaluronidase (300 units/ml) for 10 min. Perfused hearts were sliced into approximately 1–2-mm pieces, and cardiomyocytes were removed by centrifugation at 300×*g*. The small cell fraction in the supernatant was collected and expanded until 70% confluence. Cells were detached and c-kit^+^ rCPCs were isolated using Miltenyi Biotech rat anti-mouse IgG microbeads (#130-048-402) after incubating with anti-CD117 antibody (SC-5535), as per the manufacturer’s instructions.

### One-way mixed lymphocyte reaction

The immunogenicity of rCPCs was evaluated by co-culturing rCPCs with rat splenocytes. Splenocytes were isolated from spleens harvested from WKY and Brown Norway (BN) rats. Briefly, spleens were removed aseptically and placed in tissue culture dishes containing 5 ml media and minced with scalpels. Splenocytes were isolated by mechanical dissociation, and the cell suspension was filtered through a cell strainer (100 μm). Erythrocytes were lysed by incubating the cell suspension with red blood cell lysis solution for 3 min at room temperature. To evaluate immune responses to allogeneic and syngeneic rCPCs, mitotically inactivated WKY rat CPCs were cultured with CFSE-labeled WKY or BN lymphocytes in a 1:5 ratio. hCPCs were co-cultured with BN rat lymphocytes to investigate the xenogeneic response. After co-culturing for 5 days, T cells were isolated, and proliferation was assessed by measuring CFSE intensity after gating for CD3^+^ cells. The stimulation indexes for proliferation of human and rat lymphocytes were calculated by comparing the fold change of individual alloreactive and xeno-reactive lymphocyte proliferation to the mean of syngeneic lymphocyte proliferation.

### MI and cell transplantation

To evaluate the in vivo immunogenicity and cardiac repair potential of allogeneic CPCs, CPCs were transplanted into the ischemic region of the heart, as described previously [8]. Briefly, immunologically divergent rats (6–8 weeks old) underwent left thoracotomy under isoflurane (2%) anesthesia and myocardial infarction (MI) was induced by permanent ligation of the left anterior descending (LAD) coronary artery using 6–0 sutures. After confirming ischemia by visual inspection, 1 million rat or human CPCs per animal (5 million cells/kg, corresponding to 1 million cells on an average of 200 g rat) suspended in basal medium (IMDM) were injected in the peri-infarct region in 4 equal doses. Injection of IMDM alone served as a vehicle control. To determine the extent of rCPC engraftment, rCPCs overexpressing GFP were used in some experiments. A total of 6 experimental groups were used to achieve desired combinations: for allogeneic evaluation, BN female rats received: (I) IMDM, or (II) WKY rCPCs; for syngeneic evaluation, WKY female rats received, (III) IMDM, or (IV) WKY rCPCs; for xenogeneic evaluation, BN Female rats received, (V) IMDM, or (VI) hCPCs.

### Preparation of cell free medium and quantification

At the end of the reaction, supernatant was collected and precleared of cellular debris and particulate matter by centrifugation at 1000*g* for 30 min, followed by 20,000*g* for 30 min. Protein content was quantified using the bicinchoninic assay (BCA) method (Thermofisher, Waltham, MA). To normalize the protein content, we used the following formula: (concentration factor) × (total volume of medium)/(total protein content of supernatant medium). ELISA was performed for in the core facility at the University of Maryland School of Medicine using ELISA kits (Millipore and R&D systems Inc. Billerica, MA), according to the manufacturer's protocol.

### Echocardiography

Two-dimensional and M-mode echocardiography was performed using a VisualSonics Vevo 2100 ultrasound unit (VisualSonics, Toronto, Canada) under isoflurane (2%) anesthesia. Baseline echocardiograms were acquired on day 1 to confirm the expected reduction in cardiac function by measuring left ventricular (LV) ejection fraction (EF) and fractional shortening (FS) by blinded cardiologist. Animals with an EF of 45 ± 2% on post-operative day 1 were included in the study in order to maintain uniformity with respect to the severity of post-MI dysfunction. The similarity of post-MI dysfunction was verified in each treatment group by performing echocardiography at baseline and 24 h after MI. Following ligation of the left anterior descending artery (LAD), one million cells were injected into the LV myocardium near the ischemic area. Transthoracic M-mode images of the left ventricle in the parasternal short-axis view were obtained at the level of the papillary muscles using high-resolution M mode echocardiography. Data were calculated from 5 cardiac cycles according to the generally accepted formulas [8]. Progressive improvement in cardiac function was evaluated by repeating the echocardiography on days 7 and 28.

### Humoral immune response

The humoral immune response to allogenic, syngeneic, and xenogenic CPCs was evaluated by reactivity of rCPC-treated rat serum for alloreactive and xenoreactive anti-donor antibodies using flow cytometry. Rat and human CPCs were incubated with 50 μl of recipient or naïve serum for 30 min at 4 °C. After washing, cells were further incubated with rat anti-IgM and anti-IgG for 30 additional min followed by quantitative analysis using flow cytometry.

### Flow cytometry analysis

Heart tissue from treated animals was harvested at day 5 post-MI, minced, and then digested by collagenase D (Roche) at 37 °C for 50 min on a rocking platform (180–200 rpm). After enzymatic digestion, the cell suspension was filtered through a 70-µm cell strainer (Fisher Scientific #22363548) and centrifuged at 500×*g* for 10 min. To lyse red blood cells, the cell pellet was incubated in ammonium-chloride-potassium lysing buffer (Gibco # A10492-01) at room temperature for 3–5 min, then washed with iced cold fluorescence activated cell sorter washing buffer (2.5% fetal bovine serum in PBS without calcium and magnesium). Cells were resuspended in the washing buffer, and samples were incubated with Fc-Block (anti-rat CD16/CD32, 0.5 μg per 1 million cells) before incubation with isotype controls or primary antibodies, according to the manufacturer’s instructions. Cells were then washed with washing buffer. Approximately 2 × 10^5^ events (cells) were analyzed by flow cytometry (BD-LSRFortessa) and populations gated as detailed below and analyzed by FlowJo software. The antibodies are described in Table 1 (Data Supplement). T cells and Tregs were first gated (FSC-A vs SSC-A) as lymphocytes. For total T cells, the CD3 cells were gated and further analyzed for CD4 and CD8. For Tregs, CD4 cells were gated, from this gate, CD25^+^ and Fox-P3^+^ double-positive cells were determined. For macrophages, CD45 cells were gated. From CD45-positive cells, CD68 (Total macrophages) were gated and further analyzed for CD163 for M2 macrophages [22]. To see the uptake of rCPCs secreted GDF15 by CD48 receptor on T- cell, allogeneic rCPCs were co-cultured with spleenocytes with or without GDF15 KD for 5 days. At day 5, spleenocytes were collected and flow was performed for CD4, CD48 and GDF15.

**Table 1:** Antibody for flow cytometery

Antigen	Catalogue no.	Source	Flourochrome
C-kit	561443	BD	APC
CD90	554898	BD	PE
CD105	MA1-19594	Thermofisher	Unconjugated
CD45	561443	BD	PE
CD31	561443	BD	FITC
CD3	557030	BD	APC
CD4	561578	BD	PE-Cy7
CD8	561614	BD	V450
CD25	17-0390-82	eBioscience	APC
FoxP3	320008	Biolegend	PE
CD45	202205	Biolegend	FITC
CD45R	554881	BD	PE
CD68	MCA341A488	BioRad	FITC
CD163	NBP2-39099	Novus	AF647
CD11b/c	562222	BD	PE-Cy7
RT1A	559993	BD	PE
RT1B	56223	BD	AF647
RT1D	550982	BD	FITC
CD80	555014	BD	PE
CD86	12-0860-83	eBioscience	PE
pP65	4886	Cellsignalling	FITC
GDF15	ab39999	abcam	Unconjugated
CD48	SC-8400	SCBT	Unconjugated
CD16/CD32	553142	BD	Fc Block
Antibody for IHC			
IB4	121413	Invitrogen	AF 594
SMA	F3777	Sigma	FITC

### Histology

Tissues were processed as described previously [8]^.^ Briefly, rat hearts were excised under anesthesia after collection of echocardiographic data and perfused with 10% formalin solution, (Sigma Aldrich #HT501128). Tissues were cryopreserved using 30% sucrose (prepared in 1× PBS) and embedded in optimal cutting temperature compound (Fisher Scientific, TissueTek #NC1029572). A commercial cryostat was used to cut 7-μm sections, which were stained for different antibodies according to the manufacturers’ instructions. Tissue sections were counterstained with 4′,6-diamidino-2-phenylindole (DAPI) nuclear stain (Sigma #F6057) together with other required stains such as Foxp3 and pP65. All images were obtained with an EVOS microscope and quantified using Image J software.

### Lentivirus production and transduction

Manipulation of gene expression was performed by lentiviral transduction. All lentiviruses were produced in HEK293T. HEK293T cells (American Type Culture Collection, Manassas, VA) were cultured in DMEM media (CellGro) supplemented with 10% fetal bovine serum (Thermo Fisher Scientific #A38402-02). The lentivirus expression system from Origin (Catalog #TL710232) has 4 unique shRNA 29-mers for knockdown of GDF15. The titer of each lentivirus preparation was calculated based on the amount of virus required to yield 50% GFP^+^ cells following transduction of 100,000 rCPCs. We calculated the MOI according to the company (Origene, Inc) manufacturing protocol. Briefly, we calculated MOI using the following formula:

$$ \left( {\text{Total number of cells per well}} \right) \times \left( {\text{Desired MOI}} \right) = {\text{Total transducing units needed}}\,\left( {{\text{TU}}} \right). $$

Our desired MOI was 2. Cells were transduced in 12-well dishes with increasing amounts of lentivirus in media supplemented with 8 μg/ml polybrene (Sigma Aldrich #TR-1003). Three days after transduction, the percentage of GFP+ cells in each well were determined by flow cytometry using the LSR Fortessa. GDF15 KD was confirmed by western blot.

### Statistical analysis

Unpaired non-parametric tests with Mann–Whitney’s correction were performed to compare two groups. For comparisons between more than two groups, a one-way ANOVA with Tukey’s post hoc test was performed. Grouped echocardiography data was analyzed by 2-Way ANOVA with Bonferroni correction. Continuous data is plotted as box-and-whiskers plots. The middle horizontal line represents the median. The upper and lower whiskers represent the maximum and minimum values of non-outliers. The number of subjects is numerically expressed under each box and whisker column. Extra dots represent outliers. Data were analyzed using GraphPad Prism 9 software. P values ranging from 0.01 to 0.05, 0.01 to 0.001, 0.001 to 0.0001 or < 0.0001 is represent as significant *, very significant **, extremely significant ***/**** respectively.

## Results

### Phenotypic characterization and immunomodulatory effects of rCPCs

**Fig. 1:**
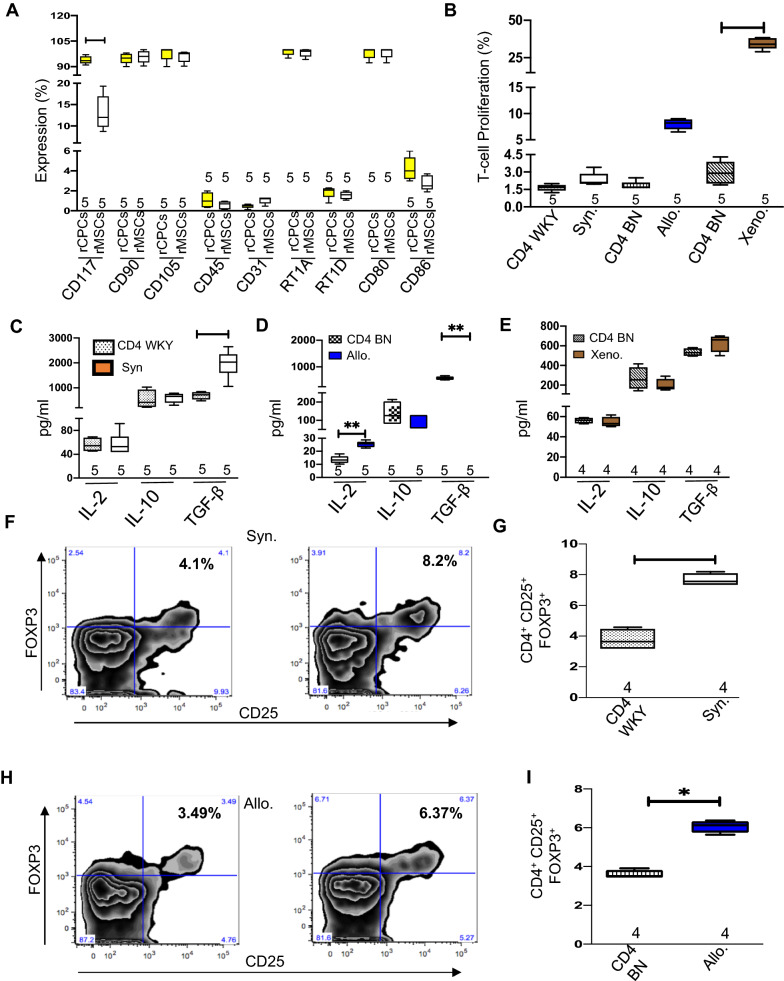
Phenotypic characterization and immunomodulatory effects of rCPCs. Rat CPCs were prepared by sorting for rat CD117 of cells prepared from the right atrial appendage of the heart of Wister Kyoto. **A** Rat CPCs and rat MSCs characterized for expression of the CD117, CD105, CD90, CD45 and CD31 markers. The expression level of RT1A, RT1D, CD80 and CD86 were measured for rCPCs and compared with rMSCs. **B** In vitro T cell proliferation in combinations of syngenic, allogenic and xenogenic cells. **C–E** Results of cytokine analysis by ELISA of syngenic and allogenic and xenogenic combinations. Percentage of T-regulatory cells (T-regs) detected by incubating rCPCs for 5 days with CD4^+^ T cells isolated from WK and BN spleens in vitro. **F**, **G** Show T-reg for syngenic and **H**, **I** illustrate T-reg in allogenic combinations. Numerical data are summarized as box and whisker plots with a median value (black bar inside box), 25th and 75th percentiles (bottom and top of box, respectively), and minimum and maximum values (bottom and top whisker, respectively). The number (n) of rats in each group indicated near the (up/below/on) each respective box and whisker plot

Consistent with our published results on human CPCs, flow cytometry also demonstrated that both rCPCs and rMSCs expressed high levels of mesenchymal markers (CD105^+^, CD90^+^), but low levels of endothelial cell markers (CD45^−^, CD31^−^) [8]. Regarding immune antigens, both cell types expressed major histocompatibility complex class I (MHC I^+^) and the co-stimulatory immune markers (CD80^+^), but not MHC II or CD86 (Fig. 1A). In addition, rCPCs expressed high levels of c-kit^+^ compared with rMSCs. These results show that CPCs display a hypoimmunogenic baseline profile, suggesting their potential to evade immune recognition in vivo. To confirm the biological relevance of the favorable baseline immunogenic profile of allogeneic rCPCs, we performed one-way mixed lymphocyte reactions with rat CD4^+^ T cells isolated from male rats of 2 different strains, WKY and BN. Rat CD4^+^ T cells were then co-cultured with rCPCs obtained from WKY rats. The combination of WKY CD4^+^ T cells and WKY rCPCs is syngeneic, whereas the combination of BN CD4^+^ T cells and WKY rCPCs is allogeneic. We also evaluated a xenogeneic combination: CD4^+^ T cells from a BN female rat with human adult CPCs. Syngeneic or allogeneic rCPCs elicited negligible T cell proliferation (Fig. 1B). Although Syngeneic CPCs didn’t elicit total T cell proliferation, allogeneic CPCs showed low (statistically insignificant) T-cell proliferation. Consistent with this low T cell proliferative response, the levels of the cytokines like transforming growth factor beta (TGF-β) was significantly increased in cell-free media of syngeneic (Fig. 1C) and interleukin 2 (IL-2) [23–26], TGF-β in allogeneic (Fig. 1D) combinations [27] as identified by multiplexed ELISA. In contrast, significant T-cell proliferation and decreased levels of TGF-β, IL-2, and IL-10 were observed in the xenogeneic co-culture conditions (Fig. 1E. Forkhead box protein 3 (FOXP3) expressing CD4+ T cells (Treg cells) are accumulated in the injured myocardium after MI injury and plays an important role to recover heart function [28–30]. To further explore the immunomodulatory properties of rCPCs, we performed in vitro co-culture studies combining rCPCs with CD4^+^ T cells isolated from WKY (syngeneic) and BN (allogeneic) rats. After 5 days in co-culture, the syngeneic (Fig. 1F, G) and allogeneic (Fig. 1H, I) combinations significantly increased the percentage of CD25^+^ and Foxp3^+^ Treg cells when compared to control. Thus, Syngenic or Allogenic CPCs do not induce an immunogenic reaction but have an immunomodulatory property by increasing a subset of CD4+ cells (T-regs). These results show that consistent with their hypoimmunogenic baseline profile, rCPCs are not immunogenic and exert immunomodulatory effects in vitro*.*

### Immunomodulatory effects of rCPCs in rat acute myocardial infarction model

**Fig. 2:**
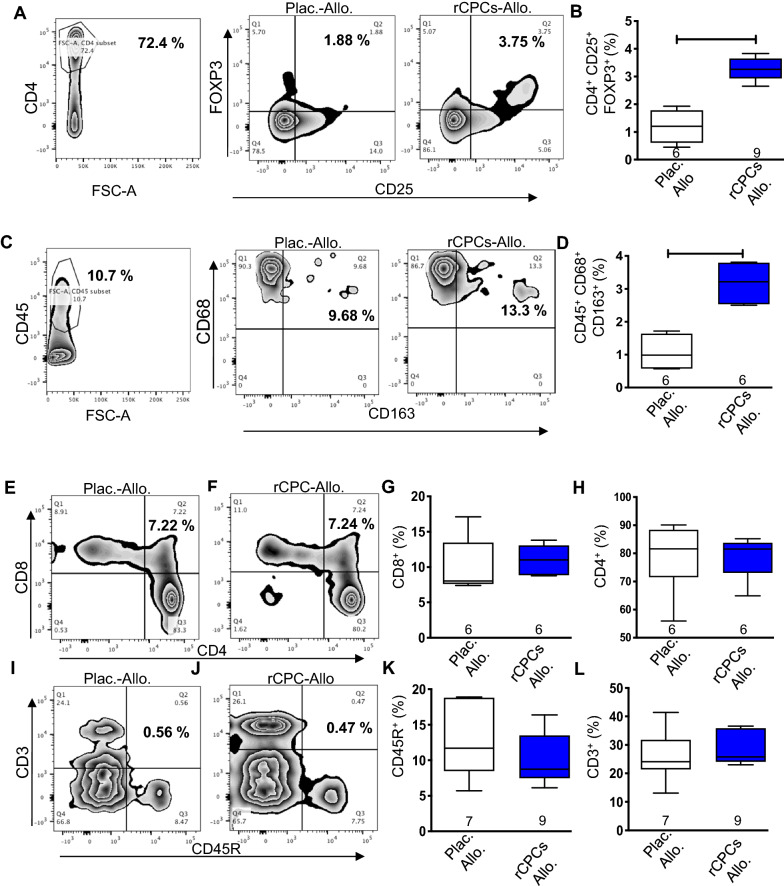
Immunomodulatory effects of rCPCs in vivo. For the in vivo study, one million rCPCs prepared from Wister Kyoto (WK) rat heats were injected intramyocardially immediately after myocardial infarctionin BN rats. Immune cells were isolated five days later. Flow images for detection of T-regs (**A**) and quantification (**B**). Flow images for M2 detection (**C**) and quantification (**D**). **E**–**H** show the percentage of CD8^+^ and CD4^+^ cells, respectively. Similarly, **I**, **J** show flow images and **K**, **L** depict the quantification of CD45R^+^ and CD3^+^ respectively. Numerical data are summarized as box and whisker plots with a median value (black bar inside box), 25th and 75th percentiles (bottom and top of box, respectively), and minimum and maximum values (bottom and top whisker, respectively). The number (n) of rats in each group indicated near the (up/below/on) each respective box and whisker plot

To determine the immunomodulatory effects of rCPCs in vivo, one million allogeneic rCPCs were injected intramyocardially in BN rats after MI and, 5 days later, the presence of T-regs in single-cell suspensions from the hearts was determined by flow cytometric analysis. Transplantation of allogeneic rCPCs induced a significant increase of CD4^+^/CD25^+^/FoxP3^+^ Tregs (Fig. 2A, B) in the injured myocardium. Since T-regs stimulate M2 polarization of monocytes/macrophages [31], we also evaluated M2 macrophages (Fig. 2C, D) in the same biological replicates. Helper T-cell (CD4^+^) and killer T-cells (CD8^+^) cells (Fig. 2E–H) or B-cells (CD45R^+^) and total T-cells (CD3^+^)cells (Fig. 2I–L) remain unchanged. Collectively, these data show that the baseline immunomodulatory profile of rCPCs correlates with immunomodulatory biological effects both in vitro and in vivo.

### Transplantation of syngeneic and allogeneic, but not xenogeneic rCPC’s improves heart function after MI

**Fig. 3:**
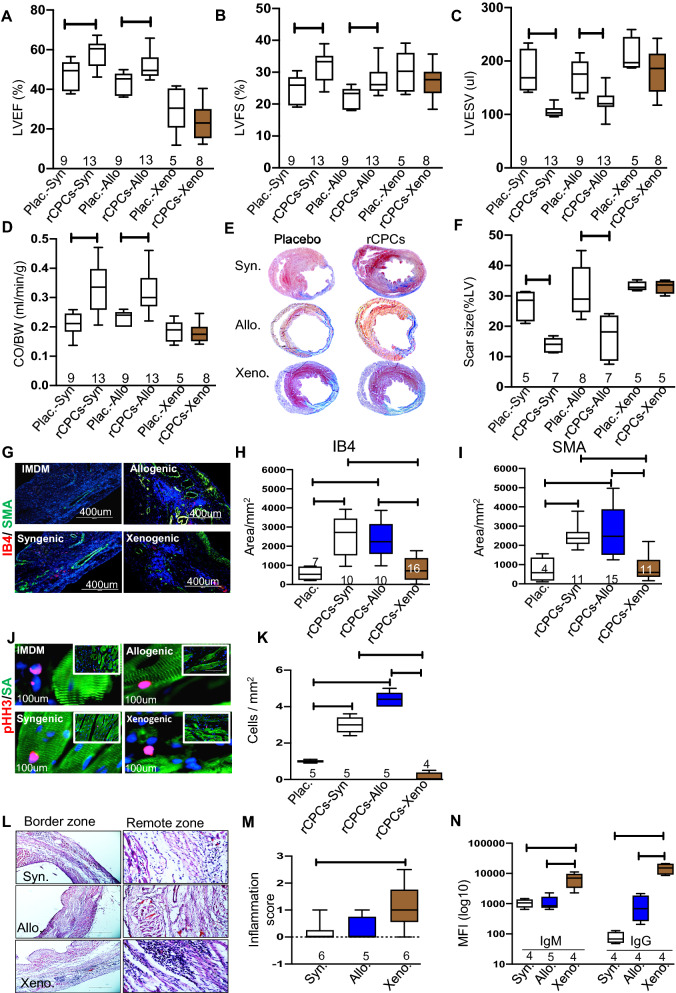
Syngeneic and allogenic, but not xenogeneic CPC transplantation improves cardiac recovery in the rat MI model. For the in vivo study, one million rCPCs prepared from Wister Kyoto (WK) rat heats were injected intramyocardially immediately after MI in Wister Kyoto (syngenic) and Brown Norway (allogenic) rats. The rats were followed for 4 weeks. Evaluation of left ventricular (LV) ejection fraction (**A**), fractional shortening (**B**), LV end-systolic volume (**C**), and cardiac output (**D**) by echocardiography on day 28. Images (**E**) and percent scar size (**F**) from Masson trichrome staining. Myocardial sections were evaluated at 28 days post-transplantation for angiogenesis by co-staining for IB4 and SMA (**G**–**I**), and cardiomyocyte proliferation for pHH3/SA (**J**, **K**). Hearts were harvested at day 28 post cell transplantation and evaluated for local inflammatory response by histology of stained sections (**L**) and inflammatory responses were scored in blinded manner (**M**). Systemic and humoral immune responses were evaluated by measuring circulating anti-donor IgM and IgG in serum (**N**). Numerical data are summarized as box and whisker plots with a median value (black bar inside box), 25th and 75th percentiles (bottom and top of box, respectively), and minimum and maximum values (bottom and top whisker, respectively). The number (n) of rats in each group indicated near the (up/below/on) each respective box and whisker plot

To determine the effects of CPCs on post-MI LV dysfunction, allogeneic, syngeneic, or xenogeneic CPCs were transplanted after MI and LV function was assessed after 28 days. Transplantation of one million allogeneic or syngeneic rCPCs significantly improved LV function after 4 weeks compared with controls (IMDM), as measured by an increase in LVEF (IMDM vs allogeneic: 42 ± 2 vs 49 ± 1%, *P* < 0.05, n = 9–12; IMDM vs syngeneic: 41 ± 2 vs 55 ± 3%, *P* < 0.05, n = 4–6) (Fig. 3A) and LV FS (IMDM vs allogeneic: 21 ± 1 vs 26 ± 1%, *P* < 0.05, n = 9–12 and IMDM vs syngeneic: 21 ± 1 vs 30 ± 2%, *P* < 0.05, n = 4–6) (Fig. 3B). No significant functional improvement of LV was observed with xenogeneic CPCs (Fig. 3A, B). In contrast to xenogeneic treatment group, LV end-systolic volume was significantly decreased after syngeneic and allogeneic rCPCs compared with IMDM and was associated with a significant increase in cardiac output after syngeneic and allogeneic rCPCs (Fig. 3C, D).

The extent of post-MI fibrosis after 4 weeks was determined by measuring the area of fibrosis (blue) relative to the total myocardial area (pink and blue) after Masson’s trichrome staining of heart sections in different groups. In contrast to xenogeneic group, heart tissues from either syngeneic or allogeneic CPCs group had significantly smaller fibrotic areas compared with placebo (Fig. 3E , F). These results indicate that, consistent with their phenotype and in vitro and in vivo immunomodulatory effects both syngeneic and allogeneic, but not xenogeneic CPCs, reduce scar size following MI in the rat.

We evaluated neovessels, arterioles formation and cardiomyocyte proliferation at day 28 post-transplantation. Transplanted allogeneic and syngeneic rCPCs significantly increased neovessel and arteriole density; confirmed by co-staining for Isolectin B4 (IB4) and smooth muscle actin (α-SMA) whereas xenogeneic rCPCs did not show any increase in neovessel and arteriole density as compared to placebo (Fig. 3G–I). Similarly, transplanted allogeneic and syngeneic rCPCs, but not xenogeneic rCPCs, significantly increased cardiomyocyte proliferation in the border zone of the infarcted area as identified by co-staining for the mitotic marker phospho-histone H3 (pHH3) and alpha-sarcomeric actin (Fig. 3J, K). The spatiotemporal development of immune rejection in the scar, border zone, and remote myocardium was examined by assessing inflammatory cells in hematoxylin and eosin-stained sections. The allogeneic and syngeneic treatment groups showed very mild cell-mediated rejection, in contrast to the xenogeneic group, which was characterized by significant mononuclear infiltration of the infarct and peri-infarct area with both interstitial and perivascular distributions (Fig. 3L, M). This interpretation was performed by a blinded cardiac pathologist. The immune scoring system applied was a modified descriptive grading system, which is a based upon the established International Society of Heart and Lung Transplantation grading system performed for heart transplantation rejection [32]. To assess the humoral response, recipient rat serum was collected at day 28 after transplantation and circulating anti-donor antibodies were detected by incubating rCPCs with the collected serum. In the allogeneic and syngeneic groups there were no significant differences compared to control, whereas in the xenogeneic group the titers of IgG and IgM antibodies were significantly increased at day 28 (Fig. 3N).

### Role of GDF15 in the effects of rCPCs on LV functional recovery

**Fig. 4:**
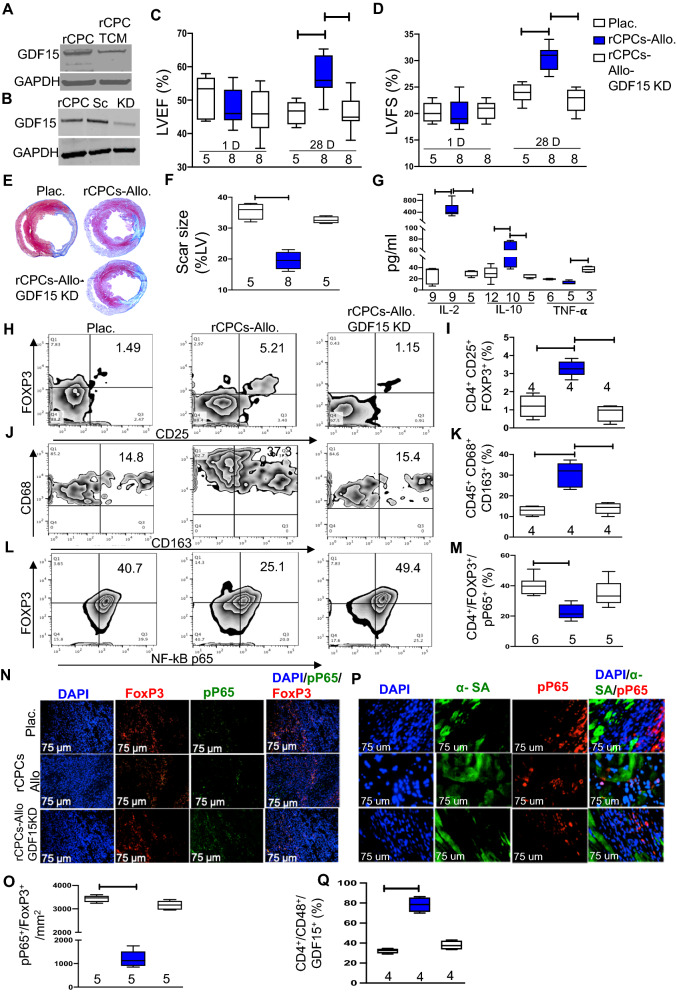
Role of GDF15 in cardiac recovery. Basal protein expression of GDF15 in rat CPCs and secretome (**A**), GDF15 protein expression in rCPCs^GDF15KD^ and rCPCs^scramble^ (**B**). Cardiac functional parameters were measured after intramyocardial injection of one million rCPCs, rCPCs^GDF15KD^ and placebo separately in the BN rat MI model. **C** LV ejection fraction, **D** LV fractional shortening. Representative picture (**E**) and quantification (**F**) of Masson trichrome staining in rat hearts. Serum ELISAs for the cytokines IL-10, IL-2, and TNF- α are shown in **G**, Single-cell suspensions of total heart lysates were used for flow cytometric analysis 5 days after intramyocardial injection of one million rCPCs, rCPCS^GDF15KD^, or placebo in BN rats. Flow images (**H**) and quantitative flow results for Tregs (**I**). Flow images (**J**) and quantitative flow results (**K**) for M2 cells. Phosphorylated NF-κB p65 flow images (**L**) and quantification (**M**). Whole hearts were also obtained on day 5 for immunohistochemistry studies. Images of phosphorylated NF-κB, FoxP3^+^, and DAPI staining (**N**) and quantification (**O**). Immunohistochemistry of phosphorylated NF-κB and sarcomeric actin with DAPI (**P**). Numerical data are summarized as box and whisker plots with a median value (black bar inside box), 25th and 75th percentiles (bottom and top of box, respectively), and minimum and maximum values (bottom and top whisker, respectively). The number (n) of rats in each group indicated near the (up/below/on) each respective box and whisker plot. **Q** Invitro co-culture assay with rCPCs/ rCPCS^GDF15KD^ with BN rat spleenocytes.for 5 day and CD48 measured by FACS

Previous studies with liquid chromatography coupled with mass spectrometry (LC–MS/MS) of human CPCs have identified GDF15 as one of the highly expressed proteins in its secretome and playing an important role in modulating cell survival pathways on target cells [8]. Immunoblot analysis of the secretome of rCPCs confirmed that these cells produce significant amounts of GDF15 (Fig. 4A, B). To explore the direct role of GDF15 in myocardial functional recovery, we knocked down GDF15 expression in rCPCs (rCPCs^GDF15KD)^ and transplanted these cells in the rat MI model. Our results showed that LVEF and LVFS were significantly decreased in rats that received rCPCs^GDF15KD^ compared to control rCPCs (Fig. 4C, D). Hearts treated with rCPCs^GDF15KD^ had significantly larger scars relative to rCPCs, and scar size was significantly reduced in rCPC-treated hearts when compared with the placebo-treated group (Fig. 4E, F). Further, the levels of circulating anti-inflammatory cytokines IL-2, IL-10, were significantly lower in the rCPC^GDF15KD^ group but inflammatory cytokine Tumor Necrosis factor (TNF-a) in the rCPC^GDF15KD^ group when compared to the rCPC group (Fig. 4G). No significant difference was observed with TGF-B. To delineate the mechanism of action of GDF15, we injected 1 million rCPCs or rCPCs^GDF15KD^ intramyocardially in rats undergoing MI. To Assess the T-regs and M2 macrophages cells, single-cell suspensions from freshly obtained leukocyte-enriched fractions of the entire heart at 5 days post-MI and flow cytometry was performed. Data showed that CD4^+^/CD25^+^/FOXP3^+^ T-regs and CD45^+^/CD68^+^/CD163^+^ M2 macrphages cells were significantly decreased in rCPCs^GDF15KD^ transplanted group compared to rCPCs (Fig. 4H–K). Increased GDF15 secretion was observed with the transplantation of rCPCs as compared to rCPCs^GDF15KD^ (Additional file 1: Fig. S2), confirmed by immunohistochemistry at day 5. These data show that GDF15 expression by rCPCs plays a key role in their immunomodulatory and cardioprotective effects in vivo. Activation of NF-κB in Treg cells were observe by flow cytometry of single-cell mixtures from treated hearts (Fig. 4L) and quantify (Fig. 4M) NF-κB activation in Treg cells (CD4^+^/FOXP3^+^/pP65^+^). These results were further confirmed by immunohistochemistry by co-staining for FOXP3^+^ and pP65 for NF-κB activation in treg cells (Fig. 4N, O). NF-κB activation was decreased with rCPC treatment compared with placebo or rCPCs^GDF15KD^ treatment in the T-reg cells. There was no expression of pP65 in the cardiomyocyte (Fig. 4P). These data suggest that rCPC secretome contain GDF15, which modulates NF-kB activity in FOXP3 positive T-reg cells modulates M2 levels in the myocardium contributing to the improved myocardial recovery in rat MI model. To explore the receptor activity and downstream signaling pathways involved in the GDF15-mediated inhibition of NF-kB, we performed an invitro co-culture experiment with spleenocytes and rCPCs with or without GDF15. As the expression of Glial-cell- derived neurotrophic factor family receptor α-like (GFRAL), is highly restricted to neuronal cells of the hindbrain and is virtually absent in all of peripheral tissues [33–35]. Recently, CD48 is the first discovered receptor of GDF15 in the immune system and is exclusively expressed on immune cells [21]. In co-culture experiment we looked for the uptake of rCPCs derived GDF15 by CD48 receptor on T-cells by flow. Data showed that all CD4 cells has CD48 receptor and the uptake of GDF15 by T-cells was significantly higher in rCPCs group as compared to control and rCPC-GDF15KD cells (Additional file 1: Fig. S1). This data suggest that rCPC secretome that contain GDF15, which modulates NF-kB activity in FOXP3 positive T-reg cells by CD48 receptor present on T-cells.

### Interplay of T-regs and macrophages in myocardial functional recovery after MI

**Fig. 5:**
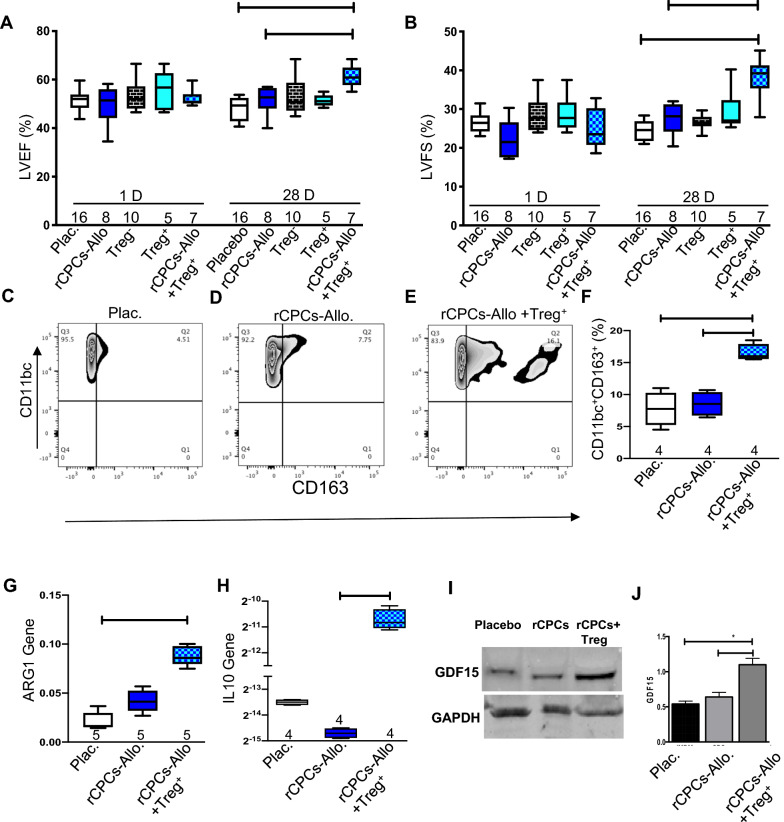
Tregs are essential for myocardial recovery after MI. Cardiac functional parameters were measured after intramyocardial injection of 1 million allogeneic rCPCs, Treg^−^, Treg^+^, rCPCs + Treg^+^ and placebo separately in a nude rat MI model. **A** and **B** represent LV ejection fraction and fractional shortening, respectively, at day 1 and day 28. Similarly, single-cell suspensions of total heart lysates were used for flow cytometric analysis of M2 elevation 5 days after intramyocardial injection of 1 million rCPCs, rCPCs + Treg^+^, or placebo. Representative images (**C**–**E**) and quantification (**F**). Left ventriculat tissue from the heart was used for RNA isolation and quantitative RT-PCR was performed to analyze the expression of **G** ARG-1, and **H** IL-10, respectively, from rCPCs, rCPCs + Treg^+^, and placebo-treated hearts. Numerical data are summarized as box and whisker plots with a median value (black bar inside box), 25th and 75th percentiles (bottom and top of box, respectively), and minimum and maximum values (bottom and top whisker, respectively). The number (n) of rats in each group indicated near the (up/below/on) each respective box and whisker plot. **F** and **G** Immunoblot analysis with quantification by densitometry revealed increased protein expression of GDF15 in nude rat myocardium after exposure to rCPCs + Treg + cells treatment relative to placebo and rCPCs (fold change relative to GAPDH listed below bands) (**I**, **J**)

To explore the possible role of Tregs in the recovery of LV function after MI, we used the nude rat which is T cell deficient. We injected 1 million cells [rCPCs, Treg^−^ cells (CD4^+^/CD25^−^), Treg^+^ cells (CD4^+^/CD25^+^), and rCPCs withTreg^+^ cells] intramyocardially following LAD ligation. Echocardiograms performed 4 weeks later showed significant increases in LV ejection fraction (Fig. 5A) and fractional shortening (Fig. 5B) in rats treated with combination cell (rCPCs + Tregs) therapy compared with rats treated with rCPCs alone or with placebo. Our data showed that rCPCs alone failed to recover the myocardium as shown in Fig. 5A and B, but when we injected rCPCs and Treg together, that significantly improved the ejection fraction and fraction shortening as compared to placebo, rCPCs, Treg^−^ cells and Treg^+^ cells. This data suggest that rCPCs derived GDF15 and Treg^+^ cells work synergistically. We also assessed M2 macrophages in single-cell suspensions of whole hearts treated with rCPCs or with the combination cell therapy (rCPCs + Treg) by flow cytometric analysis. To assess the M2 macrophages we isolated CD11b/c cells using magnetic microbeads, CD11b/c cells were co-stained for CD163 (Fig. 5C–E). CD11b/c^+^/CD163^+^ were significantly higher with the combination cell therapy compared with rCPCs therapy alone (Fig. 5F). These results suggest that Tregs augment M2 macrophage polarization, providing cardioprotective effects to the ischemic heart [18]. This concept is further supported by the gene expression of ARG1 (Fig. 5G), which is a hallmark for M2 macrophages, and the gene expression of the anti-inflammatory cytokine IL10 (Fig. 5H) in hearts that received the combination cell therapy (rCPCs + Treg). Since previous studies have shown that elevated GDF15 levels functionally protect the myocardium through an antihypertrophy response during left ventricle pressure overload, the protein levels of GDF15 and its known downstream effector, SMAD 2/3, were determined in RV myocardium [36]. We also isolated the protein from nude rat treated with placebo, rCPCs and rCPCs + Treg after MI from the infarcted zone at day 5 and determined the GDF15 expression by western blot analysis. Data showed that relative to placebo and rCPCs, rCPCs + Treg treated group has increased protein expression of GDF15 (Fig. 5I and J). This data suggests that alone rCPCs in T cell deficient rat do not secret GDF15, but in combination with Treg cells allogenic rCPCs secrete significantly higher GDF15, that co-relate to our invivo data Fig. 5A and B.

## Discussion

In this study, we have shown that allogeneic CPCs are hypoimmunogenic and immunomodulatory. Our in vitro results demonstrate that rCPCs have an allo antigenic phenotype like that of rMSCs and that they do not induce T cell proliferation. This is further supported by in vivo studies where, in contrast to xenogeneic hCPCs, allogeneic rCPCs did not induce T cell proliferation, local inflammation, or cellular or humoral immune responses. Furthermore, rCPCs appear to have immunomodulatory effects by reducing T cell proliferation, increasing Tregs, and promoting M2 macrophage polarization. Finally, allogeneic rCPCs induced significant recovery of cardiac function and reduction of scar size in rats subjected to MI. Overall, these results provide the first evidence for the immune tolerance, safety, and efficacy of allogeneic CPC therapy after MI.

We found that the expression of mesenchymal (CD90 and CD105), MHC (both I and II), and costimulatory molecules (CD80 and CD86) is similar among rCPCs and rMSCs. The lack of MHC class II expression prevents recognition by cytotoxic natural killer cells, while the low levels of expression of costimulatory molecules support escape from alloreactive CD4^+^ T lymphocytes.

T-regs, a subset of CD4+ T-cells, are necessary for cardiac repair and T-reg knock out mice have been reported to not survive beyond 4 weeks while suffering of multiple morbidities [37]. Immunomodulatory and anti-inflammatory properties of T-regs are essential for cardiac repair [38]. T-regs, through its paracrine secretion also promoting cardiomyocytes proliferation in newborns [39]. In vitro co-culturing of rCPCs in either allogeneic or syngeneic combinations did not induce significant CD3^+^ T cell proliferation compared with controls but did significantly increase Tregs (FOX-P3^+^). These in vitro results were further confirmed in vivo by flow cytometry. Allogeneic rCPCs significantly increased the population of Tregs and M2 cells but not CD8^+^ and CD4^+^ cells in the rat myocardium. These observations provide the structural basis for the hypoimmunogenic phenotype of CPCs; the functional consequences were confirmed by mixed lymphocyte reaction assays.

Allogeneic CDCs and MSCs have been shown to be efficacious in improving cardiac functional recovery [40, 41]. In fact, a recent clinical study (POSEIDON-DCM) showed that allogeneic MSCs are even more efficacious than autologous MSCs in patients with dilated cardiomyopathy [41]. We evaluated cardiac functional recovery and scar size after allogeneic, syngeneic, and xenogeneic CPC transplantation in a rat MI model. Allogeneic rCPCs improved cardiac function, as measured by LV ejection fraction and fractional shortening. Both allogeneic and syngeneic rCPCs improved cardiac function at day 28 post-transplantation compared with day 1. However, xenogeneic hCPCs failed to improve cardiac function. Histological analysis indicated that the beneficial effects of allogeneic and syngeneic cells were associated with preservation of LV wall thickness and reduction of scar size. In contrast, xenogeneic cells did not produce such effects.

Endogenous CPCs were originally thought to differentiate into cardiomyocytes, endothelial cells, and smooth muscle cells during heart development and even in the adult heart [42]. However, in more recent studies from many groups, including ours, significant differentiation of exogenous CPCs or hCPCs into cardiomyocytes has not been observed. [7, 16, 43, 44]. In the present study, fewer than 3% of exogenous CPCs differentiated into endothelial, smooth muscle, or myocardial cells (data not shown). Similar findings have been published for CDCs and MSCs [45]. Therefore, replacement of damaged cells by differentiation of CPCs cannot explain the observed beneficial effects. It is likely that CPC-derived secretory products (paracrine factors) account for the improvement in cardiac function [5]. Consistent with this notion, we recently reported that CPC secretory products promote angiogenesis and proliferation of endogenous cardiomyocytes [16, 43]. We observed similar effects in the present study using allogeneic rCPCs although there is no definitive evidence for myocyte proliferation.

Immune cells play a critical role in ischemia-induced adverse cardiac remodeling. This process can be divided into 3 distinct phases: (1) an inflammatory phase, where cardiomyocyte necrosis triggers innate immune responses, promoting infiltration of the infarcted region by neutrophils and monocytes; (2) a proliferative phase, characterized by the appearance of M2 macrophages involved in preliminary tissue stabilization by inducing processes such as angiogenesis; and (3) a final phase, characterized by the infarcted area becoming fibrotic, cardiac cells undergoing apoptosis, and the inflammatory response diminishing [46]. Modulation of immune responses during tissue remodeling is thought to be a therapeutic target for augmentation of tissue healing and repair in MI.

This study was conducted using functional readouts including immune cells in vitro and fully immunocompetent rats for the in vivo studies. We observed that even under fully immunocompetent conditions, rCPCs did not induce significant immune responses in the mixed lymphocyte reaction assays and induced minimal or no tissue infiltration of immune cells at 28 days after cell transplantation in vivo. Our results suggest that rCPCs exert immunomodulatory effects by inhibiting T cell proliferation, promoting Treg proliferation, and enhancing monocyte differentiation into M2 macrophages, all of which are typically associated with immune tolerance. These immunomodulatory effects were similar for allogeneic and syngeneic cells. Our findings are consistent with recent reports suggesting a beneficial role for Tregs in cardiac repair of the infarcted myocardium [18]. Tregs inhibit CD4^+^ and CD8^+^ T cell proliferation and inhibit the secretion of interferon gamma [47]. Additionally, Tregs play an important role in polarization of macrophages toward the M2 phenotype [48], which, in turn, plays an important role in post-infarct tissue repair [49]. Our observations support the notion that rCPCs, via regulation of T-reg cells and promotion of M2 macrophage-dependent processes, attenuate ischemia-induced adverse cardiac remodeling, preserving cardiac function. Nevertheless, further research is needed to determine whether there are additional mechanisms by which CPCs exert beneficial effects on the injured myocardium. Overall, our results clearly establish that CPCs are both hypoimmunogenic and immunomodulatory.

The most important question addressed in our study is how CPCs induce cardioprotective, hypoimmunogenic, and immunomodulatory responses. We previously showed that secreted paracrine factors influence cardiac repair/remodeling [8]. GDF15, also known as macrophage inhibitory cytokine 1, was found to be abundant in CPCs and in their secretome [8]. It has been shown previously that GDF15 enhances Treg-mediated suppression of T-cell activation by increasing IL-10 activation in Treg [17]. In this study, injection of Treg^+^ (CD4^+^ and CD25^+^) cells alone in the myocardium of nude rats (T cell–deficient) did not promote functional recovery, whereas injection of the combination of rCPCs + Treg^+^ cells promoted significant recovery, which was associated with an increase in cardioprotective M2 cells in the injured myocardium. In vivo experiments with rCPCs-GDF-15KD showed significant decreases in all cardiac parameters in immunocompetent rats, and rCPCs-GDF-15KD were unable to activate Treg and M2 cells. Furthermore, our data suggests that GDF15 secreted by CPCs inactivates NF-κB signaling in Treg cells in the ischemic myocardium, which may decrease apoptosis and increase the polarization of M2 cells. Taken together, these observations support the concept that the NF-κB/GDF15 regulatory axis in transplanted allogeneic CPCs improved cardiac function after MI by attenuating adverse cardiac remodeling and by polarizing cardioprotective M2 cells. Functional improvement in the heart was also associated with histological evidence of increased angiogenesis and cardiomyocyte proliferation. As a previous study has already demonstrated the safety of autologous CPCs in patients with chronic heart failure [6], our results support further work toward establishing banks of clinical-grade, readily-available, “off-the-shelf” allogeneic CPCs, as well as clinical trials evaluating the safety and efficacy of this cell-based approach to favorably affect the post-MI cardiac remodeling process [50, 51].

## Conclusions

Allogeneic CPCs induced minimal inflammatory responses and stimulated immunomodulatory responses by specifically increasing T-regulatory cells and M2 polarization, while maintaining their cardiac recovery potential and safety profile. Our observations strongly support the development of allogeneic CPC therapy for broader patient applications.

## Supplementary Information

**Additional file 1: Figure S1. **Invitro co-culture assay with rCPCs/rCPCs^GDF15KD^ with BN rat spleenocytes for 5 day and Flow cytometry plot gated on human CD4 T cells. CD48 measured by FACS. **Figure S2. **Whole hearts were also obtained on day 5 for immunohistochemistry studies. Images of GDF15, and DAPI staining.

## Data Availability

The datasets used and/or analyzed in this study are available from the corresponding author on reasonable request.

## Abbreviations

CPCs: Cardiac progenitor cells
hCPCs: Human cardiac progenitor cells
rCPCs: Rat cardiac progenitor cells
hMSCs: Human mesenchymal stem cells
rMSCs: Rat mesenchymal stem cells
MI: Myocardial infarction
PBMCs: Peripheral blood mononuclear cells
CFSE: 5-(and-6)-Carboxy fluorescein diacetate succinimidyl ester
IMDM: Iscove's modified Dulbecco's media
MTC: Masson TriChrome
T-reg: T-regulatory cells
GDF15: Growth differential factor 15
