# Correction: Transplanted allogeneic cardiac progenitor cells secrete GDF-15 and stimulate an active immune remodeling process in the ischemic myocardium

**DOI:** 10.1186/s12967-023-03976-0

**Published:** 2023-03-03

**Authors:** Rachana Mishra, Progyaparamita Saha, Srinivasa Raju Datla, Pranav Mellacheruvu, Muthukumar Gunasekaran, Sameer Ahmad Guru, Xuebin Fu, Ling Chen, Roberto Bolli, Sudhish Sharma, Sunjay Kaushal

**Affiliations:** 1 Department of Cardiovascular-Thoracic Surgery, Northwestern University Feinberg School of Medicine, Chicago, IL USA; 2 Department of Pediatrics, Ann & Robert H. Lurie Children’s Hospital, Chicago, IL USA; 3 Department of Surgery, University of Maryland School of Medicine, Baltimore, MD USA; 4 Division of Cardiovascular Medicine and Institute of Molecular Cardiology, University of Louisville, Louisville, USA

**Correction: Journal of Translational Medicine (2022) 20:323** **https://doi.org/10.1186/s12967-022-03534-0**

Following publication of the original article [1], we have been notified that one of the authors’ names was spelled incorrectly. It should be as follows:

First name—Xuebin, last name—Fu.
