# Supplementary material for: Transplanted allogeneic cardiac progenitor cells secrete GDF-15 and stimulate an active immune remodeling process in the ischemic myocardium
Source: J Transl Med. 2022 Jul 21;20:323. doi: 10.1186/s12967-022-03534-0 (PMC9306063; doi:10.1186/s12967-022-03534-0)
Supplement: Supplementary file 1 — Additional file 1: Figure S1. Invitro co-culture assay with rCPCs/rCPCsGDF15KD with BN rat spleenocytes for 5 day and Flow cytometry plot gated on human CD4 T cells. CD48 measured by FACS. Figure S2. Whole hearts were also obtained on day 5 for immunohistochemistry studies. Images of GDF15, and DAPI staining. [file 12967_2022_3534_MOESM1_ESM.pptx]

## Slide 1
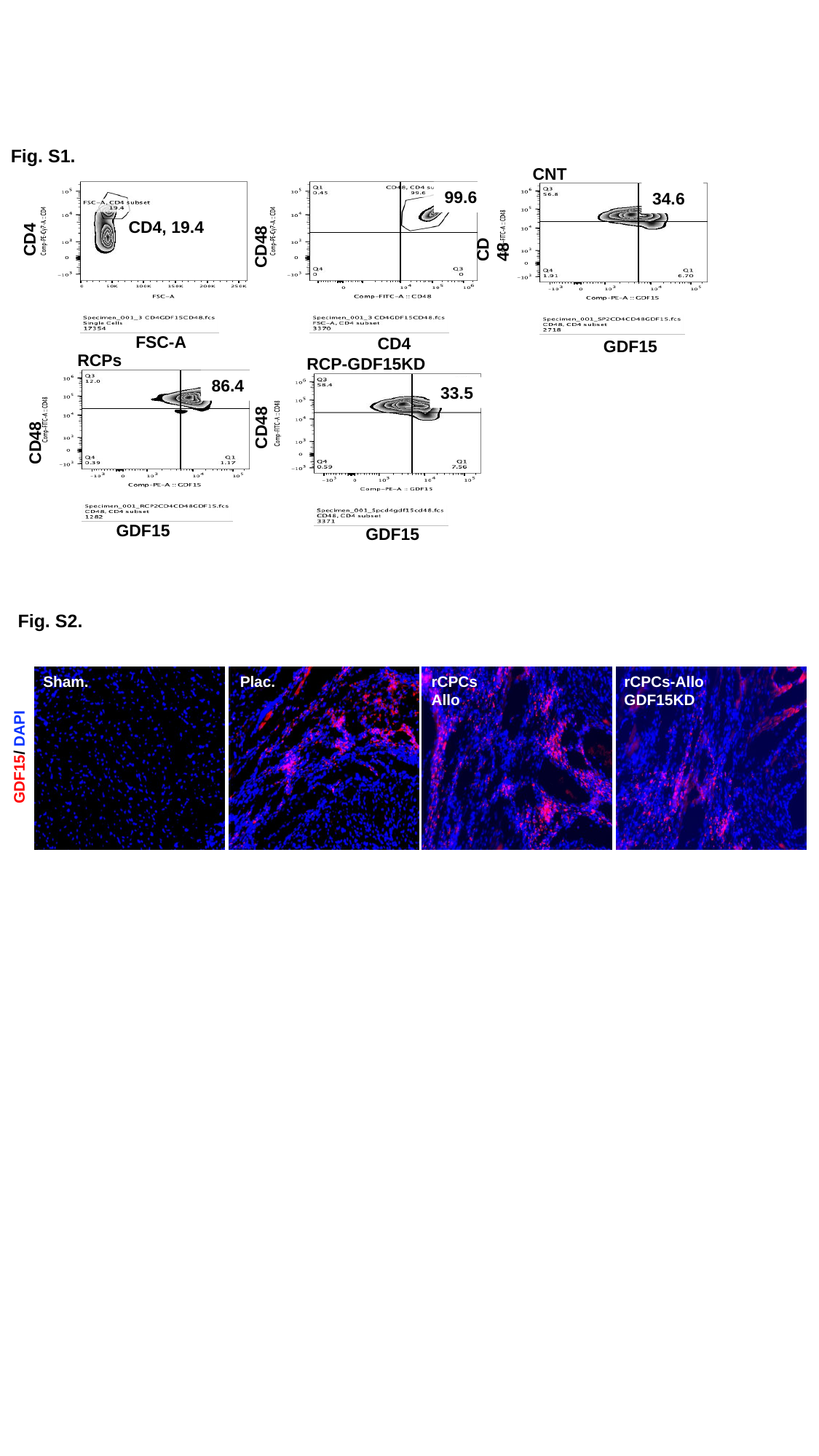

Fig. S1.
CNT
99.6
34.6
CD4, 19.4
CD48
CD4
CD48
FSC-A
CD4
GDF15
RCPs
RCP-GDF15KD
86.4
33.5
CD48
CD48
GDF15
GDF15
Fig. S2.
Sham.
Sham
Plac.
rCPCs
Allo
rCPCs-Allo
GDF15KD
GDF15/ DAPI
